# Mitochondrial Localization and Function of the Purinergic Receptor P2X_7_

**DOI:** 10.1093/function/zqab006

**Published:** 2021-01-28

**Authors:** Guido Kroemer, Alexei Verkhratsky

**Affiliations:** 1 Equipe labellisée par la Ligue contre le cancer, Université de Paris, Sorbonne Université, Inserm U1138, Centre de Recherche des Cordeliers, Paris, France; 2 Metabolomics and Cell Biology Platforms, Institut Gustave Roussy, Villejuif, France; 3 Pôle de Biologie, Hôpital Européen Georges Pompidou, AP-HP, Paris, France; 4 Suzhou Institute for Systems Medicine, Chinese Academy of Medical Sciences, Suzhou, China; 5 Department of Women's and Children's Health, Karolinska Institute, Karolinska University Hospital, Stockholm, Sweden; 6 Faculty of Biology, Medicine and Health, The University of Manchester, Manchester, UK; 7 Achucarro Center for Neuroscience, IKERBASQUE, Basque Foundation for Science, 48011 Bilbao, Spain

## A Perspective on “Mitochondrial P2X7 Receptor Localization Modulates Energy Metabolism Enhancing Physical Performance”

In a recent paper published in *Function*, Sarti et al.^1^ demonstrate previously unknown mitochondrial localization of the P2X_7_ ionotropic purinoceptor. Deletion or transgene-enforced overexpression of P2X_7_ receptor strongly influences mitochondrial morphology and function. Of note, *P2rx7*^−/−^ mice exhibit signs of dilated cardiomyopathy as well as a reduced body temperature. These findings support a novel, and somehow unexpected, role for P2X_7_ receptor in mitochondrial physiology.

The maintenance of organismal homeostasis is facilitated by the integration of regulatory circuitries. This is achieved by the pleiotropic participation of each organizational unit (such as metabolites, macromolecules, organelles, cells, and supracellular ensembles) in multiple pathways.^2^ For example, adenosine triphosphate (ATP) is not only an energy-rich metabolite but also influences the activity of kinases within cells, meaning that ATP has an intracellular signaling function. In addition, in the context of inflammation and intense physical exercise, ATP is released into the extracellular space. There, ATP and its degradation products then act on a range of metabotropic P2Y receptors (a family comprising eight genes/proteins in humans) or ionotropic P2X receptors (seven genes/proteins in humans) that are expressed in a cell type-dependent fashion to elicit a variety of adaptive responses.^3^^,^^4^ Similarly, proteins are usually multifunctional, meaning that, for example, metabolic enzymes often have signaling functions, or ion channels like cystic fibrosis transmembrane receptor may have scaffold functions affecting proteostatic networks.^2^

**Figure 1.**
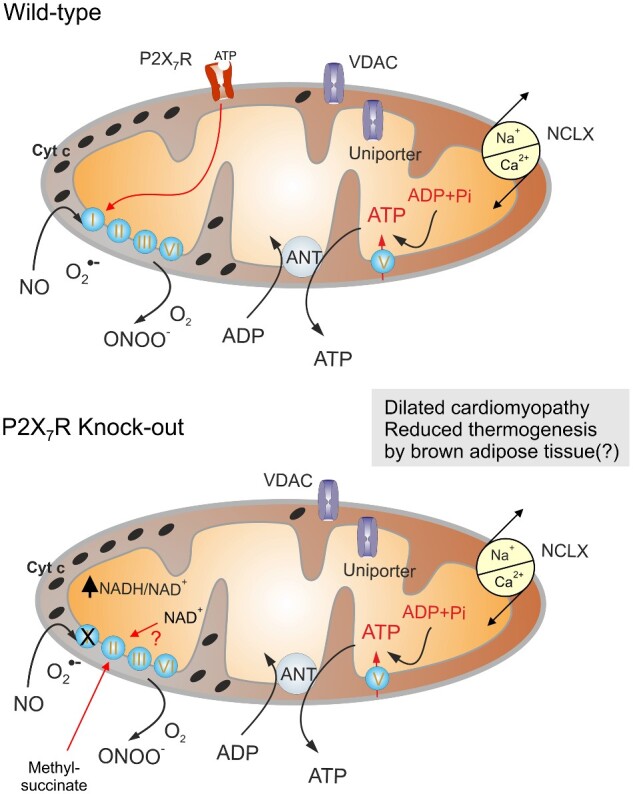
P2X_7_ Purinoceptors Regulate Mitochondrial Function. P2X_7_ purinoceptors are localized in the outer mitochondrial membrane, with the ATP-binding site facing the cytosol. These receptors are coupled with the Complex I by yet unknown cascade; when active, P2X_7_ receptors increase the expression and activity of Complex I hence increasing mitochondrial polarization, which leads to an elevation of matrix ionized Ca^2+^ and an increase in ATP production. Genetic deletion of P2X_7_ receptors leads to a decrease in Complex I activity and increased NADH/NAD+ ratio with ultimate reduction in ATP production. This is linked to cellular pathology, which underlies the development of dilated cardiomyopathy and possibly to reduced thermogenesis by the brown adipose tissue. This deficiency can be rectified by exogenous methyl-succinate which bypasses Complex I deficiency. Repletion of NAD+ possibly may have similar rescuing effect.

Sarti et al.^1^ used confocal immunofluorescence microscopy, in combination with subcellular fractionation and organelle purification techniques, to demonstrate that a significant pool of functional P2X_7_ purinoceptors is present in mitochondria, being embedded in the outer mitochondrial membrane with its ATP-binding site facing the cytosol (Figure 1). Thus, P2X_7_ receptor might sense cytosolic ATP levels to modulate mitochondrial physiology. The role of mitochondrial P2X_7_ receptors, however, extends beyond its channel function; the outer mitochondrial membrane is relatively freely permeable to ions and hence addition of another cationic conduit shall not affect mitochondrial energetic. Nevertheless, the knockout of *P2rx7* (in mouse cells) affects mitochondria profoundly. Deletion of P2X_7_ receptor decreases the mitochondrial transmembrane potential, compromises oxidative phosphorylation, increases the mitochondrial levels of the reduced form of nicotine adenine dinucleotide (NADH) levels and diminishes cellular ATP levels.^1^ This correlates with a reduction in the abundance and activity of the Complex I (NADH dehydrogenase) of the respiratory chain. Of note, the mitochondrial transmembrane potential is restored in *P2rx7*^−/−^ cells by provision of methylsuccinate, a membrane-permeant substrate of Complex II (succinate dehydrogenase) that bypasses the requirement for Complex I (Figure 1). Hence, the downregulation of Complex I is likely responsible for impaired mitochondrial function. Deletion of P2X_7_ receptor not only compromises mitochondrial function but also impairs morphology of this organelle. Cardiomyocytes of *P2rx7*^−/−^ mice contain smaller mitochondria, as determined by transmission electron microscopy. The heart in these animals exhibits anatomical signs of dilated cardiomyopathy coupled to reduced stroke volume, fractional shortening, ejection fraction, and cardiac output.^1^ A genetically induced defect of Complex I (for instance, due to knockout of *Maif1* in mice or due to its mutation in humans) causes dilated cardiomyopathy,^5^^,^^6^ suggesting that this phenotype arises from a Complex I defect. Intriguingly, supplementation of nicotinamide, which increases the levels of (oxidized) nicotine adenine dinucleotide (NAD^+^) can prevent dilated cardiomyopathy in rodent models,^7^^,^^8^ suggesting that an imbalanced NAD^+^/NADH ratio might be intrinsically pathogenic. Beyond this cardiac phenotype, Sarti et al.^1^ found that the average resting body temperature of *P2rx7*^−/−^ mice is reduced compared to wild-type controls. Reportedly, administration of nicotinamide riboside, another NAD^+^ precursor, enhances the body temperature of normal wild-type mice through an effect on brown adipose tissue.^9^ Hence, it would be interesting to investigate whether *P2rx7*^−/−^ mice have a defect in this thermogenic tissue.

Although clearly demonstrating that P2X_7_ receptors are present in or at mitochondria and they profoundly influence this organelle, the experiments reported by Sarti et al.^4^ do not clarify which of the effects of P2X_7_ receptors on whole-body physiology are mediated by its “classical” function as an ATP-gated ion channel, or by its mitochondrial localization or are mediated through its noncanonical capabilities. To resolve this issue, it would be necessary to elucidate the mechanism accounting for the active import of P2X_7_ receptors into mitochondria (or perhaps its spontaneous insertion into mitochondrial membranes) with the expectation that there is a regulatory pathway that “decides” on the subcellular destiny of P2X_7_ receptor. The finding by Sarti et al.^1^ that benzoyl-ATP (a P2X_7_ receptor agonist), hydrogen peroxide (a reactive oxygen species), and rotenone (a Complex I inhibitor) can increase receptor expression in mitochondria within a short time frame (10–60 min) points to the existence of such a regulatory mechanism. If such a pathway is unveiled in molecular detail, it might be experimentally manipulated to guide P2X_7_ receptor to exclusively mitochondrial versus nonmitochondrial localizations and to understand its local function. Alternatively, and in addition, it might be interesting to identify the local interactome of P2X_7_ receptors at mitochondria and then to block relevant protein–protein or protein–lipid reactions that occur in an organelle-specific fashion.

Notwithstanding this open conundrum, there are evolutionary arguments in favor of the notion that the intracellular function of P2X_7_ receptor may precede its function as an extracellular ATP receptor. The evolutionary history of ionotropic purinoceptors begins with eukaryotes and the very first ATP-gated channels that share trimetric structure and certain homology with mammalian P2X receptors are found in social amoeba *Dictyostelium discoideum.*^9^ The genome of amoeba contains genes encoding five subunits of ionotropic receptors, codenamed P2X_A-E_. Biophysically these receptors are archetypal ATP-gated cationic channels permeable for Na^+^, K^+^, and Ca^2+^. What makes a difference, however, is localization and function of these receptors: they are localized at intracellular organelles known as vacuoles.^10^ These organelles contribute to animal volume deregulation in response to osmotic stress, the process of vital importance because it allows adaptation to changes in the water content of the environments, something which these protists frequently experience. The *D. discoideum* is not the only species carrying intracellular P2X receptors; this trend is well conserved in evolution and P2X receptors are found in mammals in several sets of endomembranes, including lysosomes and nuclear membrane. This last discovery of mitochondrial P2X_7_ channels by Francesco Di Virgilio and Paolo Pinton’s teams further widens the biological importance of the omnipresent purinergic signaling.

## Conflict of Interest Statement

The authors declare no conflicts of interest.
